# Acute Effects of Brisk Walking on Sugary Snack Cravings in Overweight People, Affect and Responses to a Manipulated Stress Situation and to a Sugary Snack Cue: A Crossover Study

**DOI:** 10.1371/journal.pone.0119278

**Published:** 2015-03-11

**Authors:** Larissa Ledochowski, Gerhard Ruedl, Adrian H. Taylor, Martin Kopp

**Affiliations:** 1 Department of Sport Science, University of Innsbruck, Fürstenweg 185, 6020, Innsbruck, Austria; 2 Plymouth University Peninsula Schools of Medicine & Dentistry, Room N32, ITTC Building, Tamar Science Park, Derriford, Plymouth, Devon, PL6 8BX, United Kingdom; Louisiana State University, UNITED STATES

## Abstract

Research has shown that acute exercise reduces urges for chocolate in normal weight people. This study aimed to examine the effects of an acute exercise bout on urges to consume sugary snacks, affect as well as ‘psychological and physiological responses’ to stress and a ‘sugary snack cue’, in overweight individuals. Following 3 days of chocolate-abstinence, 47 overweight, sugary snack consumers were assessed, in 2 randomly ordered conditions, in a within-subject design: 15-min brisk walk or passive control. Following each, participants completed 2 tasks: Stroop color–word interference task, and handling sugary snacks. Urges for sugary snacks, affective activation and valence were assessed. ANOVAs revealed significant condition x time interaction effects for: urges to consume sugary snacks, affective valence and activation. Obtained data show that exercise reduces urges for sugary snacks and attenuates urges in response to the stress situation and the cue in overweight people.

## Introduction

The global epidemic of obesity has led to increased interest in the contribution of snacking behaviors and factors influencing self-regulatory processes [1]. Obesity has been associated with greater daily fatigue and in turn less physical activity [1]. Replacing sedentary behavior with physical activity, which increases affective valence and activation [2] and reduces psychological and physiological responses to stress [3], may help to break the habitual consumption of high energy snacks.

Failure to self-regulate high energy snacking has been likened to an addiction [4]. Food cravings have been blamed for overeating in obese individuals and for early drop out from weight loss treatments [5]. Weingarten & Elston [6] pointed out that on average 97% of women and 68% of men experience food cravings. Specific situations seem to stimulate consumption of high caloric sugary snacks. These include abstinence and associated elevation in cravings [7], stress [8,9] and negative mood [1], and exposure to cues which are associated with the past use [10–12]. Restrained eaters are particularly prone to consume energy dense food such as sugary snacks in these situations [7,13–16]. Experimental studies have shown that the intake of high caloric sugary snacks leads to an immediate reduction of emotional tension and to a decrease in tiredness in the short term, but increases both of them in the long term [17]. Consumption of sugary snacks has also been shown to increase positive affective activation and decrease negative affect [18–20].

A large body of literature has focused on the acute effects of exercise on hunger, appetite and energy intake [21], but only six studies have focused on the effects of a single session of exercise, versus passive control condition, on high energy snack food consumption, cravings and attentional bias towards food cues, among regular snackers [17,20,22–25].

It has been shown that exercise reduces chocolate cravings [25] and ad libitum snacking [24] among regular snackers who were abstinent from chocolate. In addition to that it has been shown that acute exercise attenuates physiological as well as psychological responses to stressors [26]. This fact may be an important role of exercise in attenuating mood-regulating food consumption. A study by Taylor & Oliver [25] has shown that a 15 minute bout of moderate intensity exercise, compared to rest, reduced chocolate cravings in a manipulated stress situation, and after presentation of snack foods. This study involved normal weight participants who were regular chocolate eaters, so the effects of exercise interventions on responses to a manipulated stress situation and to a sugary snack cue in people who are overweight are still unclear.

Several studies have shown a potential of single exercise bouts to improve affect and change behaviour [25,27,28]. The mechanisms of these effects are still unclear. One suggestion may be given that exercise acutely enhances affective valence and activation [29]; this in turn reduces the urge to consume stimulants to regulate mood. There is a need to find out if and how changes in affective responses to exercise are associated with changes in cravings.

Therefore, the purpose of the present study was to determine if a 15 minute bout of moderate intensity exercise reduces levels of sugary snack cravings and affect, and also attenuates increases in cravings associated with stress and cue elicited urges in overweight people.

## Materials and Methods

### Participants

After ethical approval from the “Institutional Review Board Department of Sport Sciences University of Innsbruck” participants were recruited through electronic networks (emails), via notices posted where medical and nutritional consultants worked, and through weight management support groups. To be eligible for the study, participants had to have a 25 kg/m^2^ ≥ BMI ≤ 30 kg/m^2^ and consume at least 100g of high caloric sugary snacks, such as chocolate, per day. Forty-seven participants (29 women) with a mean (SD) age of 28.11 (9.55) years took part in this counterbalanced, within-subject, randomised study on two separate days (between one and seven days apart).

### Measures

**Urges for sugary snacks.** The State Food Craving Questionnaire [FCQ-S] [30–33] adapted for sugary snacks, was used to assess cravings for sugary snacks. With a total of 15 items (3 items per dimension), this questionnaire assesses five dimensions of food craving: (1) An intense desire to consume food, (2) anticipation of positive reinforcement that may result from eating, (3) anticipation of relief from negative states and feelings as a result of eating, (4) possible lack of control over eating if food is eaten, (5) craving as hunger/ physiological state. Participants should indicate on a 5-point scale from 1 (strongly disagree) to 5 (strongly agree) the extent to which they agreed with each statement (item). Information about validity and reliability for the FCQ-S has been provided previously by [30] and [33].

**Affect and Perceived Activation.** Affective valence was evaluated by the Feeling Scale [FS] [34]. This 11 point single item scale ranges from-5 (very bad) to +5 (very good). Perceived activation was assessed by the Felt Arousal Scale [FAS] [35]. This 6 point single item scale ranges from 1 (low arousal) to 6 (high arousal). These two scales have been widely used to capture the acute affective responses to exercise [29], compared with passive control conditions, and target basic affect as a dimensional Circumplex model of affect [2]. As a domain-generic measure it is based on a strong theoretical and empirical rationale [36,37]. The generic nature of the measure is gaining in importance, given the dual context of emotional food cravings and exercise [25]. Convergent validity information for FS and FAS has been provided previously [38].

**Physiological measures.** Systolic (SBP) and diastolic blood pressure (DBP) and heart rate (HR) were assessed (OMRON RX Classic II) with the participant in a semi–recumbent seated position, and a BP cuff placed on the wrist of the non–dominant arm. Participants wore a Fingertip Pulsoximeter (PC-60B) throughout the walking session.

### Task and Procedure

To elevate sugary snack cravings, participants were told to abstain from eating sugary snacks (such as chocolate) for three days and they were asked to abstain from eating, drinking anything (except water) and exercising for two hours prior to each assessment.

Upon arrival at the laboratory, all participants signed an informed consent prior to their involvement in the experiment. To identify potential contraindications against moderate intensity exercise, participants completed the Physical Activity Readiness Questionnaire [PAR- Q] [39]. For the attendance at both sessions, participants were paid 20 Euros.

**Exercise session.** The active condition started with a two minute warm-up, followed by a 15 minute semi self-selected brisk walk on a flat treadmill. Participants were told to walk briskly, as if they were trying to catch a bus, but not up to the point of breathlessness. This instruction has been used effectively in other studies to ensure walking at a moderate intensity [23–25,40]. Additionally, to determine exercise intensity, a Rating of Perceived Exertion (RPE) using the 6 (extremely light)- 20 (extremely hard) Borg Scale [41,42] was recorded every 2 minutes. Heart rate was recorded every minute.

**Passive session.** The passive control condition involved sitting quietly in the laboratory for 15 minutes. No reading material was provided and participants were spoken to only when measures were administered.

**Stress induction and trigger situation (cues).** Following both conditions, participants were told to sit quietly for 5 minutes. Afterwards they completed two tasks, with a 5 minute rest period between the tasks. Initially participants had to do a computerized Stroop test [43]. This task has been shown to elevate physiological arousal [44] and was used successfully in previous studies to elicit stress [25]. Then, the participants were offered a selection of high caloric sugary snacks. They were asked to unwrap one sugary snack of their choice and handle it for about 30 seconds, without eating it. This trigger situation was used effectively in the study of Taylor & Oliver [25] to increase craving for sugary snacks.

FCQ–S, FS and FAS were administered during both sessions at seven time points: pre treatment, mid treatment, post treatment, pre Stroop, post Stroop, pre Trigger and post Trigger. SBP and DBP were assessed at pre treatment, pre Stroop, post Stroop, pre Trigger and post Trigger.

### Statistical Analyses

Data were analyzed using the statistical programme SPSS 18.0. Sample size calculations were performed with G*Power 3.1.2., using data from Taylor & Oliver [25], which reported that post-exercise FCQ-S was reduced by a mean (95% confidence interval for mean difference: effect size) of 6.00 (2.48–9.52: 0.57) for chocolate cravings. Sample size calculations indicated that 35 participants for each condition were required to detect a significant difference between the exercise conditions versus the control condition on urges for sugary snacks, the primary dependent variable in the present study (α = 0.05, 95% power).

First, related t-tests were used to identify any differences between baseline measures in the exercise and the control condition. If there were any significant differences, between baseline measures, data were transformed by the amount of calculated difference. As a manipulation check, we examined the effects of the stress task on heart rate. To calculate the effects of exercise on cravings for sugary snacks, fully repeated measures ANOVAs were performed to examine the main and interaction effects of condition (two levels: exercise and control) and time (three levels: pre treatment, mid treatment, post treatment) for the FCQ–S total score. To assess the responses to both tasks (stress situation and trigger situation) additional repeated measures ANOVAs were performed to identify main and interaction effects of condition (two levels: exercise and control) and time (two levels: pre stroop, post stroop [stress] and pre trigger, post trigger [trigger]) for the FCQ–S total score, for SBP and DBP. To assess the effects of exercise on self-reported pleasure and activation a series of fully repeated measures ANOVAs were performed to examine the main and interaction effects of condition (two levels: exercise and control) and time (pre treatment, mid treatment, post treatment). To examine affective responses to both tasks (stress situation and trigger situation) further repeated measures ANOVAs were used to identify main effects of condition (two levels: exercise and control) and time (two levels: pre stroop, post stroop [stress] and pre trigger, post trigger [trigger]) for FS and FAS. Mauchly´s Test of Sphericity was conducted initially, followed by ANOVAs, with Greenhouse—Geisser correction where appropriate.

## Results

At the baseline assessment the participants show a mean (SD) BMI of 27.63 (2.62) kg/m^2^. The mean SBP/ DBP in the exercise and control conditions (before treatment) was 128.71 (12.99)/ 83.63 (10.43) mmHg and 128.24 (11.60)/ 80.73 (12.30) mmHg. The mean heart rate during brisk walk and rest was 117.12 (15.92) BPM and 76.40 (13.79) BPM. The mean self-selected intensity of exercise was subsequently calculated as fairly light [RPE = 10.52 (2.39)].

Table 1 presents mean (SD) scores for study variables (FCQ-S, FS, FAS) over time. Table 2 shows means (SD) for HR, SBP and DBP over time.

**Table 1:** Mean (SD) scores for Food craving Questionnaire State (FCQS), Feeling scale (FS) and Felt Arousal Scale (FAS) by condition over time.

	time						
	Pre treatment T1, x¯ (S.D.)	During treatment T2, x¯ (S.D.)	Post treatment T3, x¯ (S.D.)	Pre stroop T4, x¯ (S.D.)	Post stroop T5, x¯ (S.D.)	Pre trigger T6, x¯ (S.D.)	Post trigger T7, x¯ (S.D.)
**FCQS—total**							
Exercise	36.89 (10.39)	28.36 (7.96)	30.06 (7.92)	32.04 (8.32)	35.89 (8.63)	33.55 (8.74)	38.32 (9.65)
Control	38.89 (9.91)	39.87 (10.42)	38.57 (9.36)	38.49 (9.42)	40.57 (8.95)	38.77 (9.60)	42.15 (10.16)
**FS**							
Exercise	2.43 (1.51)	3.40 (1.25)	3.38 (1.30)	2.87 (1.62)	2.74 (1.17)	2.74 (1.21)	2.70 (1.18)
Control	2.36 (1.78)	2.51 (1.84)	2.49 (1.83)	2.49 (1.83)	2.47 (1.77)	2.45 (1.80)	2.34 (1.71)
**FAS**							
Exercise	2.55 (1.04)	3.06 (0.97)	2.81 (0.90)	2.38 (0.87)	2.89 (1.03)	2.40 (0.88)	2.72 (0.93)
Control	2.55 (1.04)	2.15 (1.27)	2.11 (1.32)	2.09 (1.30)	2.57 (1.40)	2.13 (1.31)	2.36 (1.29)

**Table 2:** Effect of exercise on systolic blood pressure (SBP), diastolic blood pressure (DBP) and heart rate (HR); reactions to a stress situation and a trigger, by condition over time.

	Pre stroop	Post stroop	Pre trigger	Post trigger
**SBP (mmHg)**				
Exercise	129.30 (14.96)	128.62 (13.98)	127.38 (15.91)	129.02 (12.87)
Control	125.37 (12.29)	127.20 (15.77)	128.91 (13.17)	130.70 (12.97)
**DBP (mmHg)**				
Exercise	84.57 (12.61)	82.38 (11.83)	80.85 (12.36)	84.57 (11.09)
Control	84.20 (13.93)	85.02 (15.68)	85.83 (15.85)	88.70 (16.84)
**HR**				
Exercise	79.38 (15.17)	85.17 (17.40)	79.66 (15.26)	85.17 (15.71)
Control	74.38 (13.63)	76.85 (12.89)	74.51 (13.30)	76.62 (11.73)

Initial manipulation checks using repeated measures ANOVAs showed that there were significant main effects of time for HR, in response to both the stressor (F(1, 92) = 17.865, p< 0.01, eta^2^ = 0.163) and sugary snack cue (F(1, 92) = 30.548, p< 0.01, eta^2^ = 0.249) and a significant main effect of condition (two levels: exercise and control) for stressor (F(1, 92) = 5.244, p< 0.05, eta^2^ = 0.054), thereby indicating that the experimental manipulations did indeed increase measures of physiological arousal.

**Fig 1:**
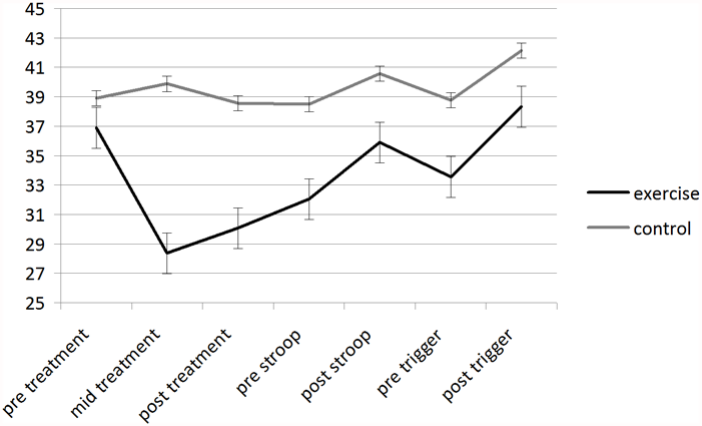
Mean (SEM) scores for State Food Craving Questionnaire for each condition over the whole duration of the session.

**Effects of an acute exercise bout on sugary snack cravings.** Fully repeated measures ANOVAs revealed a significant condition (two levels: exercise and control) by time (three levels: pre treatment, mid treatment, post treatment) interaction (F(2, 184) = 59.416, p<0.01; eta^2^ = 0.392) for total FCQ–S (as shown in Fig. 1). Post hoc t tests showed significant between condition differences in self-reported sugary snack craving at: mid treatment (p<0.01) and post treatment (p<0.01). T tests also revealed significant reductions in cravings between pre treatment and mid treatment (p<0.01), post treatment (p<0.01) in the exercise condition but no change in the control condition.

**Craving—responses to a stress situation following an acute exercise bout.** Repeated measures ANOVAs revealed significant condition (two levels: exercise and control) by time (two levels: pre stroop, post stroop) interaction (F(1, 92) = 15.992, p< 0.01, eta^2^ = 0.148), main effect for time (F(1, 92) = 180.697, p< 0.01, eta^2^ = 0.663), and main effect of condition (F(1, 92) = 9.446, p< 0.01, eta^2^ = 0.093) for total FCQ–S (as shown in Fig. 1).

**Craving—responses to a trigger situation following an acute exercise bout.** Repeated measures ANOVAs revealed no significant condition (two levels: exercise and control) by time (two levels: pre trigger, post trigger) interaction (F(1, 92) = 2.989, p = 0.087, eta^2^ = 0.031) for total FCQ–S. But there was a main effect of time (F(1, 92) = 103.769, p< 0.01, eta^2^ = 0.530) and condition (F(1, 92) = 5.489, p< 0.05, eta^2^ = 0.056) for total FCQ–S (as shown in Fig. 1).

**Fig 2:**
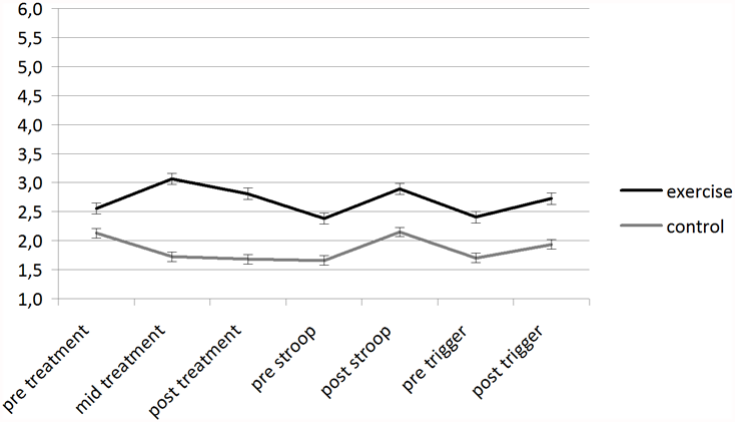
Mean (SEM) scores for Felt Arousal Scale for each condition over the whole duration of the session.

**Fig 3:**
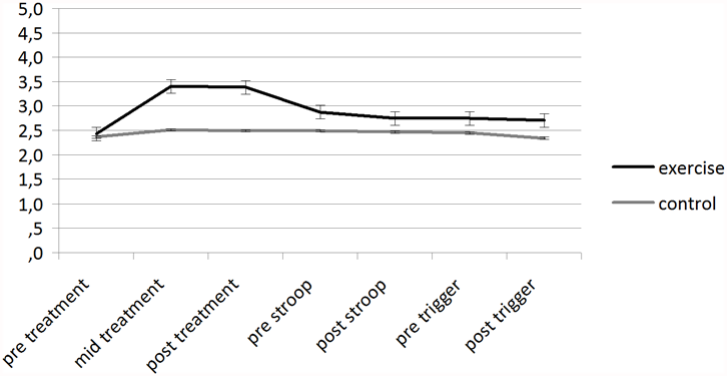
Mean (SEM) scores for Feeling Scale for each condition over the whole duration of the session.

**Effects of an acute exercise bout on affect.** Fully repeated measures ANOVAs revealed a significant condition (two levels: exercise and control) by time (three levels: pre treatment, mid treatment, post treatment) interaction (F(2, 184) = 31.475, p<0.01; eta^2^ = 0.255) for FAS (as shown in Fig. 2). Post hoc t tests showed significant differences in self-reported activation at mid treatment (p<0.01) and post treatment (p<0.01). Fully repeated measures ANOVAs revealed a significant condition (two levels: exercise and control) by time (three levels: pre treatment, mid treatment, post treatment) interaction (F(2, 184) = 32.347, p<0.01; eta^2^ = 0.260) for FS (as shown in Fig. 3). Post hoc t tests showed significant differences in affective valence at mid treatment (p<0.01) and post treatment (p<0.01).

**Affective—responses to a stress situation following an acute exercise bout.** Fully repeated measures ANOVAs revealed a significant main effect of time (two levels: pre stroop, post stroop) for FAS (F(1, 92) = 84.820, p<0.01; eta^2^ = 0.480) (as shown in Fig. 2) and FS (F(1, 92) = 4.696, p< 0.01, eta^2^ = 0.049) (as shown in Fig. 3).

**Affective—responses to a trigger situation following an acute exercise bout.** Repeated measures ANOVAs revealed significant main effect of time (two levels: pre trigger, post trigger) for FAS for sugary snack cue (F(1, 92) = 32.058, p< 0.01, eta^2^ = 0.258) (as shown in Fig. 2); Post hoc t tests showed significant differences in self-reported activation at: post trigger (p<0.05). Repeated measures ANOVAs revealed significant main effect of time (two levels: pre trigger, post trigger) for FS for sugary snack cue (F(1, 92) = 4.619, p< 0.05, eta^2^ = 0.048) (as shown in Fig. 3).

**Physiological—responses to a trigger situation following an acute exercise bout.** Repeated measures ANOVAs revealed only a significant main effect of time for DBP for sugary snack cue (F(1, 91) = 11.907, p< 0.01, eta^2^ = 0.116).

## Discussion

Findings from this study show that a 15-min walk of moderate intensity reduces craving for high-calorie sugary snacks, in an overweight sample. Our results are in agreement with those of investigations in which reduced craving for high-calorie snacks [20,23–25] were observed to occur after physical activity, in normal weight people. This study also demonstrates that opening and handling sugary snacks as well as the exposure to a stress situation increased high energy food cravings, but exercise attenuated these responses. So the findings of this study support the idea that a single bout of exercise can reduce cue or stress elicited cravings. These findings are similar to those reported by Taylor & Katomeri [26] for cigarettes.

### Reactions to a manipulated stress situation

Subjects reacted to an increased level of stress with an increased pulse rate, which is in agreement with previous findings [26]. In contrast to the Taylor & Katomeri study [26], however, we could not observe any rise in blood pressure as a consequence of being confronted with stress. Analysis of the blood pressure values over different time points yielded no clearly interpretable picture. Craving for sugary snacks increased after exposure to stress in overweight individuals. Similar results were reported for normal weight individuals [25]. As far as affect is concerned, we found a general deterioration in affective well-being (lowering of affective valence and increase of affective activation) in overweight subjects exposed to stress. It should be noted that the artificially induced stress situation with the help of the Stroop Interference Task can function as a model for simulating only acute stress, but it has to be considered that many people experience chronic stress in their life. Therefore, the potential of short bouts of physical activity on coping with chronic stressors need to be examined in future studies.

### Reactions to a trigger situation

Opening a bag of sugary snacks and keeping it open resulted in a marked increase in the pulse rate. Exposure to high-calorie food stuff also caused a marked increase in self-rated craving. This result is in agreement with Taylor & Oliver [25]. Overweight people thus do not seem to differ in their response (physiological as well as subjectively assessed craving) to trigger situations from persons with normal weight. Future studies are necessary to determine the extent to which craving for sugary snacks, subjectively assessed, leads to consumption of sugary snacks and whether overweight persons differ from persons with normal weight in this respect.

The present study also revealed a significant increase in affect during and immediately after physical activity in comparison to the passive control condition. The present study was not powered to conduct mediation analysis and further research is needed to determine if change in affect does indeed mediate the effects of exercise on sugary snack craving. According to Thayer [1], a boost of energy while feeling lethargic or fatigued leads to consumption of high-calorie food.

The present study had some limitations. Considering strength and weaknesses of our study we would like to underline the use of recommended measurements (FS, FAS, FCQ-S) at a total of 7 time points. However, we think the FCQ-S for assessing craving for sugary snacks is not quite suitable for repeated use because of the 15 items in the scale. In view of the similarity between several items in this scale, repeated use of this instrument led to boredom and a lack of willingness to answer the questions carefully and veritable in some participants. For future investigations with repeated assessments we recommend to shorten this questionnaire after performing a factor analysis. While the present study replicated the findings of other studies that have shown an acute effect of exercise on affect, compared with a passive control condition, other measures of affect (e.g. Activation- Deactivation Adjective Checklist [45]) and cravings (eg, visual analogue scale: 0–100) should be used in further research to confirm the present findings. Another limitation of this study is the absence of a measure of trait cravings and the absence of a measure of physical activity levels; therefore it is not easy to compare the obtained results with other studies [24,25]. Furthermore, in this study it was not possible to verify the mandated abstinence from sugary snacks and the two—hour fast. Future investigations could use a glucometer or self-reported diary to ensure that participants observe the study conditions.

To avoid examiner-induced effects, it would be useful to reduce the level of interaction between subject and tester with the help of technical aids such as touch screens in future studies. Employment of additional objective methods for gathering data on cravings (e.g. eyetracker or fMRI) would be of interest in future studies.

### Practical implications

This study adds to the increasing evidence that physical activity can somehow help to regulate the urge to consume snack food. It may be easy for overweight people to fit in short bouts of low-moderate intensity physical activity, instead of being sedentary, to elevate affective activation and valence and reduce high energy food cravings which may be triggered by stress and the presence of snack foods.

## Conclusion

Short bouts of physical activity may reduce the craving for sugary snacks in overweight people. Concurrent positive changes in affect suggest that further research is needed to examine the possible mediating role of affect (valence and activation) in the effects of physical activity on cravings. When snacking has become habitual and poorly regulated by overweight people the promotion of short bouts of physical activity could be valuable for reducing the urge to consume at times when the person may be particularly vulnerable such as during stress and when snack foods are available.
